# Identification of Druggable Cancer Driver Genes Amplified across TCGA Datasets

**DOI:** 10.1371/journal.pone.0098293

**Published:** 2014-05-29

**Authors:** Ying Chen, Jeremy McGee, Xianming Chen, Thompson N. Doman, Xueqian Gong, Youyan Zhang, Nicole Hamm, Xiwen Ma, Richard E. Higgs, Shripad V. Bhagwat, Sean Buchanan, Sheng-Bin Peng, Kirk A. Staschke, Vipin Yadav, Yong Yue, Hosein Kouros-Mehr

**Affiliations:** Department of Oncology, Eli Lilly and Company, Indianapolis, Indiana, United States of America; National Cancer Center, Japan

## Abstract

The Cancer Genome Atlas (TCGA) projects have advanced our understanding of the driver mutations, genetic backgrounds, and key pathways activated across cancer types. Analysis of TCGA datasets have mostly focused on somatic mutations and translocations, with less emphasis placed on gene amplifications. Here we describe a bioinformatics screening strategy to identify putative cancer driver genes amplified across TCGA datasets. We carried out GISTIC2 analysis of TCGA datasets spanning 14 cancer subtypes and identified 461 genes that were amplified in two or more datasets. The list was narrowed to 73 cancer-associated genes with potential “druggable” properties. The majority of the genes were localized to 14 amplicons spread across the genome. To identify potential cancer driver genes, we analyzed gene copy number and mRNA expression data from individual patient samples and identified 40 putative cancer driver genes linked to diverse oncogenic processes. Oncogenic activity was further validated by siRNA/shRNA knockdown and by referencing the Project Achilles datasets. The amplified genes represented a number of gene families, including epigenetic regulators, cell cycle-associated genes, DNA damage response/repair genes, metabolic regulators, and genes linked to the Wnt, Notch, Hedgehog, JAK/STAT, NF-KB and MAPK signaling pathways. Among the 40 putative driver genes were known driver genes, such as *EGFR*, *ERBB2* and *PIK3CA*. Wild-type *KRAS* was amplified in several cancer types, and *KRAS*-amplified cancer cell lines were most sensitive to *KRAS* shRNA, suggesting that *KRAS* amplification was an independent oncogenic event. A number of MAP kinase adapters were co-amplified with their receptor tyrosine kinases, such as the FGFR adapter *FRS2* and the EGFR family adapter *GRB7*. The ubiquitin-like ligase *DCUN1D1* and the histone methyltransferase *NSD3* were also identified as novel putative cancer driver genes. We discuss the patient tailoring implications for existing cancer drug targets and we further discuss potential novel opportunities for drug discovery efforts.

## Introduction

Recent advancements in DNA sequencing technology have enabled the sequencing of whole cancer genomes and identification of commonly mutated, amplified, and deleted genes across cancer types. The Cancer Genome Atlas (TCGA) effort was set up to sequence and analyze several thousand individual cancers, giving a snapshot to disease-specific genetic backgrounds and cancer drivers [1]–[6]. Integrated analysis of TCGA datasets identified 127 significantly mutated cancer-associated genes representing distinct biological pathways and cellular processes [6]. The average number of driver mutations per tumor sample was two to six, suggesting that a small number of mutated driver genes could induce carcinogenesis [6]. In breast cancers, only three genes (*GATA3*, *PIK3CA*, and *TP53*) were found to be mutated at >10% incidence across all patient tumors. Further analysis revealed pathway-specific genetic driver mutations in breast cancer subtypes, such as *BRCA1/2* alterations and *PIK3CA* alterations in basal-like and luminal breast cancers, respectively [4]. In colorectal cancers, twenty-four genes were commonly mutated and most of the genes mapped to the Wnt, TGF-b, PI3K, p53 and RAS signaling pathways [3]. In lung cancers, eleven genes were commonly mutated, including *TP53*, oxidative stress response genes and squamous differentiation genes [1]. These studies have shed light into the major genetic drivers of cancer subtypes and have also identified potentially druggable pathways linked to these subtypes. The advancements will accelerate drug development by offering novel patient tailoring strategies for pathway-specific inhibitors. However, the TCGA studies have mostly focused on mutations and rare translocations, with less attention placed on gene amplifications in cancers. Since gene amplification is an important mechanism of carcinogenesis, we sought to mine the TCGA datasets to identify novel targets and drivers amplified across cancer types.

Gene amplification in cancer cells provides a means for overexpression of cancer-promoting driver genes, such as *EGFR* and *ERBB2* on chromosomes 7 and 17, respectively. Gene amplification occurs somatically in a restricted region of the cancer genome through various mechanisms, such as breakage-fusion-bridges cycles [7]. These amplified regions, known as amplicons, can span kilobases to tens of megabases and can include multiple oncogenic genes as well as passenger genes in the amplified regions [8]. The length of amplicons can vary substantially based on the genomic locus and cancer type. For example, single gene amplification of *KIT* on chromosome 4 can occur in testicular tumors [9], yet larger amplicons containing *KIT*, *PDGFRA*, and *KDR* are amplified in glioblastoma [10]. Because amplicons often contain many genes, including passenger genes not related to oncogenesis, it is often difficult to identify the cancer driver gene(s) responsible for the amplification. Strategies to identify the cancer genes driving an amplicon include mapping the minimal region of amplification (MRA) across many tumor samples, identifying positive correlation between copy number and mRNA expression of genes, and experimental validation with siRNA/shRNA knockdown in cells. Such analyses have to date identified amplified genes with a demonstrated role in carcinogenesis [7]. However, most analyses to date have relied on small samples sizes, which result in large MRAs and potential false positive genes. The TCGA datasets offer a unique collection of tumor samples with large sample sizes to identify amplified cancer driver genes in distinct cancer types.

Here we describe a bioinformatics screening strategy to identify potentially druggable cancer driver genes amplified across TCGA datasets. We used GISTIC2 analysis of TCGA datasets (cBio portal) and identified 461 genes that were statistically amplified in two or more TCGA datasets comprising 14 cancer types. Genes with putative or verified roles in cancer were identified using Cancer Genes cBio database. We assigned a druggability score for each gene by integrating data from four external druggability indices. From the 461 genes, we identified 73 potentially druggable amplified genes with a known or putative role in carcinogenesis. We then used correlation analysis with copy number and mRNA expression data from several thousand TCGA patient samples to identify potential cancer driver genes among the list. This resulted in the identification of 40 putative cancer driver genes linked to diverse oncogenic processes, including epigenetic regulators, cell cycle-associated genes, DNA damage response/repair genes, metabolic regulators, and genes linked to the Wnt, Notch, Hedgehog, JAK/STAT, NF-KB and MAPK signaling pathways. The putative cancer driver activity was further validated by accessing the shRNA hairpin activity in cancer cell lines using the Project Achilles database [11]. Additional validation was performed on a subset of the genes using siRNA/shRNA knockdown in cancer cell lines containing the gene amplification of interest. Among the 40 putative driver genes were known driver genes, such as *EGFR* and *ERBB2*, as well as novel targets, such as *DCUN1D1* and *NSD3*. *KRAS*, a prominent cancer driver with known activating mutation in cancer [12], was found to be amplified in a subset of ovarian, gastric, lung, and uterine cancers. We discuss the implications for drug discovery efforts and we identify novel patient tailoring strategies for existing therapeutic targets.

## Materials and Methods

### Bioinformatics analysis

TCGA datasets from 14 cancer subtypes were analyzed for gene amplification using the GISTIC2 algorithm in the cBio portal (http://www.cbioportal.org). The 14 cancer subtypes include BLCA - Bladder Urothelial Carcinoma, BRCA - Breast invasive carcinoma, CRC – Colorectal Cancer (COAD and READ studies combined together), GBM - Glioblastoma multiforme, HNSC - Head and Neck squamous cell carcinoma, KIRC - Kidney renal clear cell carcinoma, LGG - Brain Lower Grade Glioma, LUAD - Lung adenocarcinoma, LUSC - Lung squamous cell carcinoma, OV - Ovarian serous cystadenocarcinoma, PRAD - Prostate adenocarcinoma, SKCM - Skin Cutaneous Melanoma, STAD - Stomach adenocarcinoma, and UCEC - Uterine Corpus Endometrioid Carcinoma. Genes that were amplified in two or more TCGA studies were pooled together to make a list of 461 genes. Level 3 SNP6 and RNAseq version 2 data were retrieved from TCGA website, and level 3 SNP6 data were further mapped to gene level using R package CNTools. Pearson correlation coefficients for gene copy number (SNP6) versus gene expression (RNASeq) were calculated for genes of interest using function cor() in R. The data analysis code in R and GAWK can be provided upon request. Each gene was assigned a druggability score based on data from the external databases Ensembl, InterPro-Blast, BioLT-Drugbank and Qiagen Druggability list. For each database, a gene was given a 0–4 druggability score, with 0 being undruggable and 4 being an established drug target. A gene with a “1” druggability score in any of the four databases was considered “potentially druggable” and included in the final gene list. The gene list was also uploaded to the Cancer Genes database (cBio portal) and genes linked to oncogenesis were included in the final gene list.

### Project Achilles

The Project Achilles database consists of shRNA depletion scores from a pooled genomic library tested across a panel of cancer cell lines [11]. We developed a method to score gene dependency in each cell line by weighting each hairpin according to the degree of consistency with other hairpins designed against the same gene, in a manner similar to that described by Shao et. al [13]. We reasoned that if tumor cell lines varied in their dependency on a particular driver gene, then hairpins effectively targeting that gene should give similar shRNA depletion scores in the dependent lines. We calculated pairwise correlations of depletion scores across the panel for all hairpins from the group of shRNA constructs designed to target a particular gene. Then each shRNA was weighted by the number of other shRNAs from the gene set that were highly correlated to it (Spearman correlation coefficient is larger than 0.35 with a p-value<0.01). A gene-level composite score (shRNA score) was then obtained by weighted summation of the shRNA depletion scores. These gene dependency profiles were used to calculate likelihood ratio scores for the association of gene mutation or copy number with shRNA sensitivity by comparing the gene mutation model to a “null model” (without any gene mutation).

### Cells

Cells were obtained from American Type Culture Collection (ATCC) and were grown in Dulbecco's modified Eagle's medium (DMEM) media supplemented with 10% fetal bovine serum. Amplified and non-amplified cell lines were chosen for each cancer amplified gene of interest. For each cancer amplified gene, the cell lines used for validation studies and their corresponding gene copy numbers are as follows: (1) *NSD3*: H1581 (7 copies), H1703 (6 copies), SW48 (5 copies), SW837 (non-amplified); (2) *DCUN1D1*: KYSE (6 copies), T47D (4 copies), SW48 (non-amplified), HCT15 (non-amplified). Copy number values were obtained from published CCLE datasets [14].

### Gene knockdown

For gene knockdown genes, we used shRNA lentiviral transduction particles purchased from Sigma (Mission, SHCLNV). *DCUN1D1* shRNA constructs were TRCN0000133666, TRCN0000134440, TRCN0000134715, TRCN0000136858, and TRCN0000137482. For *NSD3* knockdown studies, we used On-Targetplus SMARTpool siRNA targeting human Nsd3 (Thermo Scientific). Cells were infected with lentiviral shRNA particles at multiplicity of infection (MOI) ranging from 5–10 in the presence of 10 ug/ml polybrene. siRNA/shRNA experiments were carried out according to established protocols [15].

### Cell based assays

Antibodies used for western blot analysis include rabbit anti-DCUN1D1 (Sigma, HPA035911), rabbit anti-WHSC1L1 (Proteintech, 11345-1-AP). Western blot was carried out according to conventional protocols. Cell proliferation and apoptosis assays were performed with the Cell Titer Glo and Caspase Glo assays (Promega) according to manufacturer instructions. Cell cycle analysis was performed with propidium iodide staining of cancer cell lines using conventional protocols [15].

## Results

### Identification of gene amplifications in TCGA datasets

**Figure 1:**
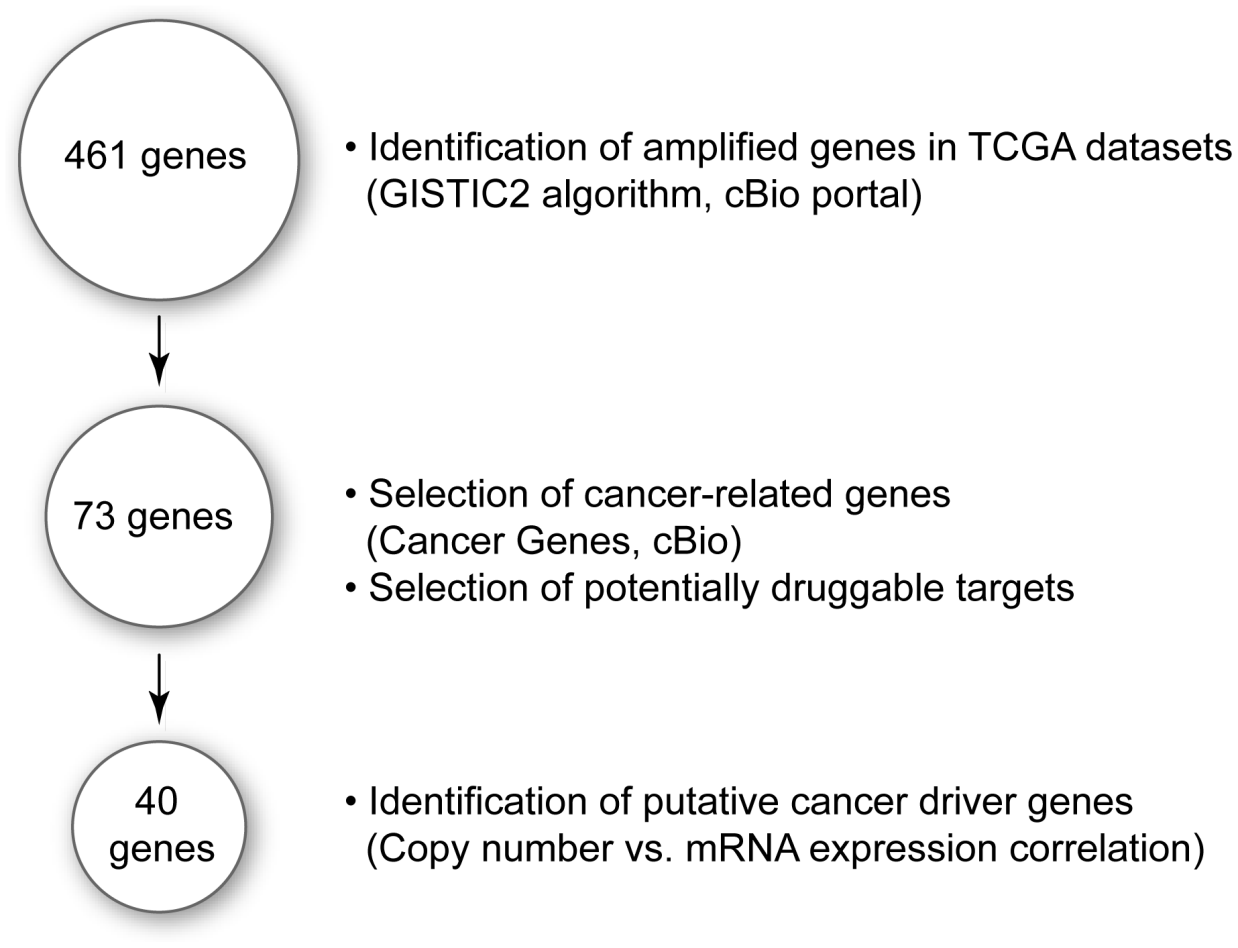
Flowscheme for the identification of cancer amplified genes with putative cancer driver activity. TCGA datasets were mined for gene amplification (GISTIC2 analysis, cBio portal) and 461 gene amplifications were identified. The list was narrowed to 73 genes cancer-related genes that were potentially “druggable” based on external druggability databases. From the 73 genes, 40 putative cancer driver genes were identified based on copy number versus mRNA expression analysis of TCGA data.

TCGA datasets comprising 14 cancer types were analyzed with GISTIC2 algorithm (cBio portal) to identify gene amplifications in patient tumor samples. Genes were scored for statistical likelihood of amplification, and those genes showing amplification in two or more datasets were identified (Figure 1). A total of 461 genes were identified as potentially amplified genes (Table S1). In some cases, several genes (e.g., *CD274* and *NDUFC2*) were amplified in two or more datasets that originated from a single cancer subtype (Figure 1, Table S1). The gene list was further narrowed by identifying the subset of genes with established or putative roles in oncogenesis as well as genes that were potentially druggable. First, the gene list was cross-referenced with the Cancer Genes database (cBio portal), which showed that less than 25% of the 461 genes were linked to oncogenesis. Next, the genes were assigned a druggability score based on the druggability indices from four external databases (Ensembl, InterPro-Blast, BioLT-Drugbank and Qiagen Druggability list). For each database, a gene was given a 0–4 druggability score, with 0 being undruggable and 4 being an established drug target. A gene with a “1” druggability score in any of the four databases was considered “potentially druggable” and included in the final gene list. From the analysis, a total of 73 potentially druggable cancer amplified genes were identified across the TCGA datasets (Figure 1).

**Figure 2:**
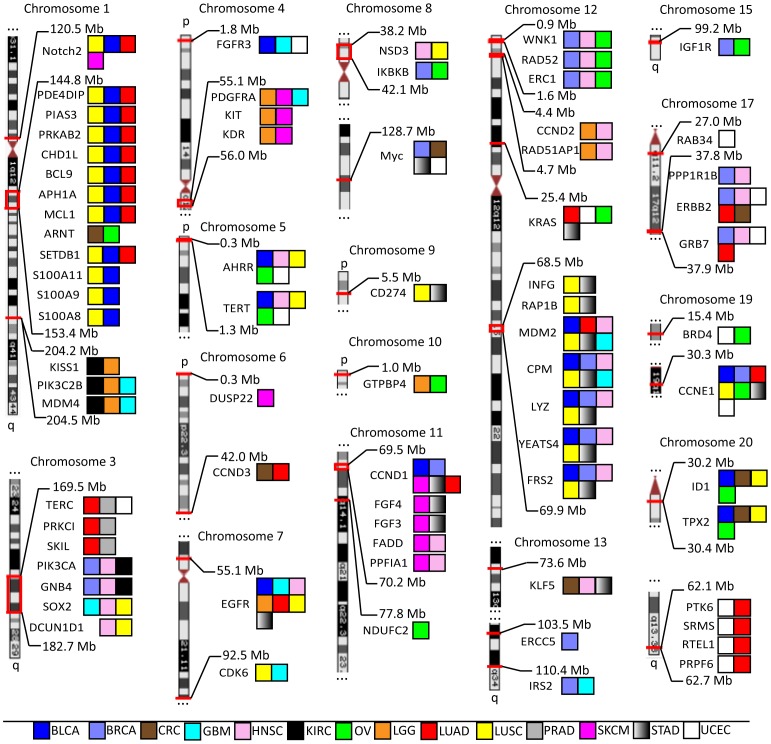
Identification of 73 genes amplified in TCGA datasets. From the initial list of 461 genes amplified in one or more TCGA datasets, 73 amplified genes were identified with potentially “druggable” properties as well as established/putative roles in oncogenesis. Genes/amplicons are arranged by chromosomal location, with their genomic location marked as shown (Mb = Megabase). Colored boxes indicate cancer types with TCGA designations, as follows: BLCA - Bladder Urothelial Carcinoma, BRCA - Breast invasive carcinoma, CRC – Colorectal Cancer (COAD and READ studies combined together), GBM - Glioblastoma multiforme, HNSC - Head and Neck squamous cell carcinoma, KIRC - Kidney renal clear cell carcinoma, LGG - Brain Lower Grade Glioma, LUAD - Lung adenocarcinoma, LUSC - Lung squamous cell carcinoma, OV - Ovarian serous cystadenocarcinoma, PRAD - Prostate adenocarcinoma, SKCM - Skin Cutaneous Melanoma, STAD - Stomach adenocarcinoma, UCEC - Uterine Corpus Endometrioid Carcinoma.

The 73 cancer amplified genes were located across the genome and the majority of the genes clustered in disease loci (Figure 2). Of the 73 genes, 57 genes clustered in 14 loci across the genome and the remaining 18 genes were focal amplifications. Within a cluster, the genes tended to be amplified in similar cancer types. For example, a chromosome 20q cluster comprising four genes (*PTK6*, *SRMS*, *RTEL1*, and *PRPF6*) were all amplified in uterine/endometrial cancers and lung adenocarcinomas. A chromosome 1q cluster contained 12 genes, such as *SETDB1*, *BCL9*, *PIAS3*, and *MCL1*, and 11 of the 12 genes were amplified in lung squamous cancers and bladder cancers (Figure 2). A well-studied cluster on chromosome 4q containing *PDGFRA*, *KIT*, and *KDR* was amplified in glioma and melanomas [10]. Because of the stringency used in Gistic2 analysis, we likely underestimated the cancer types in which a gene amplification occurred. Therefore it is likely that the 73 cancer genes we identified were amplified in additional cancer types not represented here (Figure 2).

Among the 73 cancer amplified genes were a number of established drug targets, such as *EGFR*, *ERBB2* and *KIT* (Figure 2). *ERBB2* on chromosome 17 was amplified in 5 cancer types and was co-amplified with the MAP kinase adaptor *GRB7* and *PPP1R1B*. *EGFR* on chromosome 7 was amplified as a single gene in 7 cancer types, validating the importance of this drug target in cancer [16]. The list also included a number of targets currently in clinical development across the industry, such as *CDK6*, *PIK3CA*, *PIK3C2B* and *NOTCH2*. *CDK6* on chromosome 7q was amplified as a single gene in lung squamous cancer and glioblastoma, while *PIK3CA* resided on a chromosome 3q cluster with 6 other genes and was amplified in multiple cancer types (Figure 2) [17]. Several previously validated cancer amplified genes, such as *FAK*/*PTK2*, were not identified in the analysis, in part due to high stringency that was applied to the bioinformatics analysis to reduce false positive hits [18].

### Identification of amplified cancer genes with putative cancer driver activity

**Figure 3:**
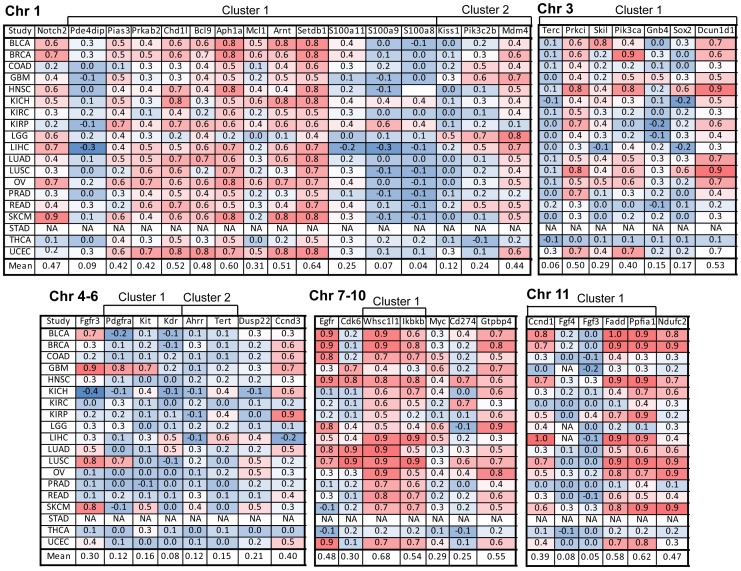
Gene copy number and mRNA expression correlation analysis to identify putative driver genes amplified on chromosomes 1–11. Pearson correlation coefficients were calculated by analyzing gene copy number and mRNA expression from individual patient-derived samples in TCGA datasets. Shown are the correlation coefficients for each TCGA cancer subtype and the mean correlation across all cancer types (red denotes high correlation, blue denotes low correlation). Abbreviations of TCGA datasets are listed in Figure 1.

**Figure 4:**
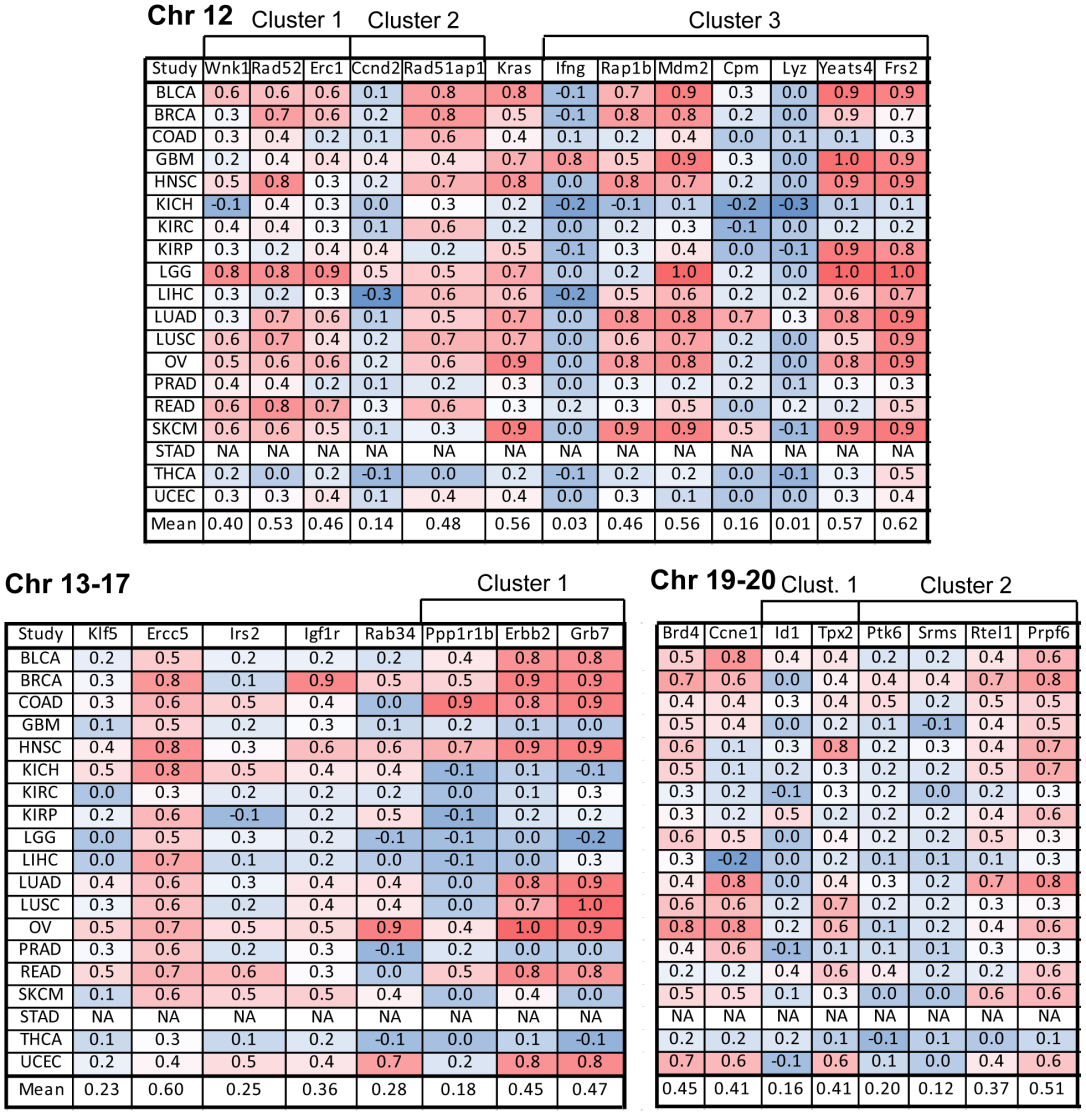
Gene copy number and mRNA expression correlation analysis to identify putative driver genes amplified on chromosomes 12–20. Pearson correlation coefficients were calculated by analyzing gene copy number and mRNA expression from individual patient-derived samples in TCGA datasets. Shown are the correlation coefficients for each TCGA cancer subtype and the mean correlation across all cancer types (red denotes high correlation, blue denotes low correlation). Abbreviations of TCGA datasets are listed in Figure 1.

Because some of the genes identified as cancer amplified genes may be passenger genes in the amplicons, we further analyzed the gene set to identify putative cancer driver genes. This was done by calculating the Pearson correlation coefficient between copy number and mRNA expression value from TCGA patient tumor data. Correlation coefficients were calculated for each of the 14 cancer types and the average correlations across all cancer types were calculated (Figures 3–4). The analysis revealed a wide range of copy number versus mRNA expression correlations for the genes. Putative cancer driver genes were expected to show high copy number versus mRNA expression correlation. Validated cancer drivers such as *ERRBB2*, *EGFR*, and *KRAS* demonstrated high copy number versus mRNA expression correlation in the corresponding cancer types they regulate (*ERBB2* r = 0.9 in breast cancer, *EGFR* r = 0.8 in lung adenocarcinoma, *KRAS* r = 0.9 in ovarian cancer) (Figure 3–4).

The copy number versus expression analysis revealed the potential driver genes that were amplified in the gene clusters. For example, the chromosome 1q cluster with 12 amplified genes contained 4 genes with copy number vs. expression correlation greater than 0.5 (*SETDB1*, *ARNT*, *APH1A*, and *CHD1L*), suggesting that these may be the driver genes in the amplicon (Figure 3). Among the 12 genes, *SETDB1* showed the highest overall correlation, consistent with recent reports that *SETDB1* is a cancer amplified gene with demonstrated driver activity [19], [20]. The other three genes may also play potentially significant roles in carcinogenesis – *APH1A* is a gamma secretase complex subunit in the Notch pathway, *ARNT* is a subunit in the HIF1 complex, and *CHD1L* is a DNA helicase in the DNA damage response pathway [21]. Four genes in the amplicon displayed copy number versus expression correlation less than 0.3 (*PDE4DIP*, *S100A11*, *S100A9*, and *S100A8*) (Figure 3). The chromosome 3 cluster with 7 genes contained 2 genes with copy number versus expression correlation greater than 0.5 (*DCUN1D1* and *PRKCI*) and 4 genes with copy number versus expression less than 0.3 (*TERC*, *SKIL*, *GNB4*, and *SOX2*). *PRKCI* is a serine/threonine kinase in the NF-KB pathway and previous tissue microarray data validated this gene as a potential novel cancer driver gene [22]. *DCUN1D1* is an E3 ubiquitin ligase complex subunit with potential cancer driver activity, which we further validated with shRNA knockdown (below). While *PIK3CA* displayed an overall correlation coefficient 0.4, it displayed high correlation in breast cancer (r = 0.9), head and neck squamous cancer (r = 0.8), and uterine/endometrial cancers (r = 0.7) (Figure 3).

The chromosome 11q cluster contained 5 genes, including *CCND1*, a well-established cell cycle regulator and oncogenic driver. While *CCND1* displayed high copy number versus expression correlations in liver cancer (r = 1.0), bladder cancer (r = 0.8), lung squamous cancer (r = 0.7), head and neck caner (r = 0.7) and breast cancer (r = 0.7), the correlations were lower in other cancer types, suggesting that *CCND1* amplification is a disease-specific oncogenic driver (Figure 3). Two other genes in the amplicon, *FADD*, and *PPFIA1*, displayed higher overall correlation across cancer types, implicating these genes as potential novel cancer drivers for further investigation. *FADD*, an apoptotic effector molecule, was previously identified as a novel cancer driver gene in a panel of 167 laryngeal/pharyngeal cancers, warranting further investigation into its mechanism of oncogenesis [23]. It is important to note that correlation of mRNA expression to copy number is not essential in principle for a gene to be a cancer driver gene. Therefore, genes with low mRNA expression versus copy number correlation are not necessarily passenger genes. For example, the chromosome 1q cluster contained *MCL1*, a gene with a cancer driver signature based on Project Achilles (data not shown) but with a mean mRNA expression versus copy number correlation of 0.31.

To identify the amplified cancer genes with highest overall cancer driver activity, we ranked the genes in order of highest copy number versus mRNA expression correlation across all cancer types. We identified 40 genes with overall r greater than 0.3 (Table 1). The r = 0.3 cutoff was used because several genes demonstrated high r in a small number of cancer types. For example, *FGFR3* displayed r>0.7 in four cancers (bladder cancer, glioblastoma, lung squamous, and melanoma) but r<0.5 in other cancers. Similarly, *CDK6* demonstrated r>0.7 in only 4 cancers (glioblastoma, head and neck cancer, lung adenocarcinoma, and lung squamous cancer) while *IGF1R* had r>0.7 in only one cancer (breast cancer) (Figure 3–4). Among the 40 genes with highest cancer driver activity, the top two most highly ranked genes were *NSD3*/*WHSC1L1* and *SETDB1*, two important histone methyltransferases (Table 1). While *SETDB1* was recently established as a bona fide amplified cancer driver in melanoma and lung cancer [19], [20], the role of *NSD3*/*WHSC1L1* has not been well characterized and so we further validated its oncogenic role in vitro (below). Two other chromatin regulators, the chromatin reader Brd4 and histone acetyltransferase *YEATS4*, were also highly ranked as putative cancer driver genes. Other gene families that were represented in the list include Notch pathway genes (*NOTCH2*, *APH1A*), metabolic regulatory genes (*NDUFC2*, *PRKAB2*), Hedgehog pathway genes (*DCUN1D1*), Wnt pathway genes (*BCL9*), NF-KB pathway genes (*ERC1*, *PRKCI*, *IKBKB*), JAK/STAT pathway genes (*PIAS3*), MAPK signaling effectors (*KRAS*, *FRS2*, *GRB7*), receptor tyrosine kinases (*FGFR3*, *EGFR*, *ERBB2*, *IGF1R*), DNA damage response/repair genes (*RAD51AP1*, *RTEL1*, *ERCC5*, *RAD52*, *CHD1L*), p53-associated genes (*MDM2*, *MDM4*, *GTPBP4*), and cell cycle regulatory genes (*CCNE1*, *TPX2*, *CCND3*, *CDK6*) (Table 1).

**Table 1:** Identification of cancer amplified genes with high copy number versus expression correlation.

Gene name	Entrez ID	Chr	r	Description
*NSD3/WHSC1L1*	54904	8	0.68	Histone methyltransferase
*SETDB1*	9869	1	0.64	Histone methyltransferase
*PPFIA1*	8500	11	0.62	LAR protein-tyrosine phosphatase-interacting protein (liprin)
*FRS2*	10818	12	0.62	Adapter protein (FGFR/MAPK signaling)
*APH1A*	51107	1	0.6	Gamma-secretase complex subunit (Notch pathway)
*ERCC5*	2073	13	0.6	DNA endonuclease (DNA excision repair)
*FADD*	8772	11	0.58	Apoptotic adaptor, candidate driver oncogene
*YEATS4*	8089	12	0.57	NuA4 histone acetyltransferase (HAT) complex subunit
*MDM2*	4193	12	0.56	E3 ubiquitin-protein ligase linked to p53
*KRAS*	3845	12	0.56	GTPase, proto-oncogene
*GTPBP4*	23560	10	0.55	GTPase, negative regulator of p53
*IKBKB*	3551	8	0.54	Serine kinase (NF-KB pathway)
*DCUN1D1*	54165	3	0.53	E3 ubiquitin ligase complex subunit, candidate oncogenic driver
*RAD52*	5893	12	0.53	DNA damage repair enzyme
*CHD1L*	9557	1	0.52	DNA helicase involved in DNA damage response
*ARNT*	405	1	0.51	HIF1 complex subunit
*PRPF6*	24148	20	0.51	Spliceosome component (U4/U6-U5 tri-snRNP complex)
*PRKCI*	5584	3	0.5	Serine/threonine kinase (NF-KB pathway)
*BCL9*	607	1	0.48	Trancriptional regulator linked to Wnt signaling
*EGFR*	1956	7	0.48	Receptor tyrosine kinase
*RAD51AP1*	10635	12	0.48	DNA damage response/repair gene
*NOTCH2*	4853	1	0.47	Type 1 transmembrane protein (Notch pathway)
*NDUFC2*	4718	11	0.47	NADH dehydrogenase (Complex I) accessory subunit
*GRB7*	2886	17	0.47	Adapter protein (EGFR/MAPK pathway)
*ERC1*	23085	12	0.46	Regulatory subunit of IKK complex (NF-KB pathway)
*RAP1B*	5908	12	0.46	GTPase, Ras oncogene family
*ERBB2*	2064	17	0.45	Receptor tyrosine kinase
*BRD4*	23476	19	0.45	Epigenetic regulatory gene, chromatin reader
*MDM4*	4194	1	0.44	Nuclear protein, negative regulator of p53
*PIAS3*	10401	1	0.42	Small ubiquitin-like modifier (SUMO) ligase (JAK/STAT)
*PRKAB2*	5565	1	0.42	AMP-activated protein kinase (AMPK) subunit
*CCNE1*	898	19	0.41	Cell cycle regulator (G1-S)
*TPX2*	22974	20	0.41	Spindle assembly factor, linked to Aurka
*PIK3CA*	5290	3	0.4	Phosphoinositide-3-kinase (PI3K) subunit
*CCND3*	896	6	0.4	Cell cycle regulator (G1-S)
*WNK1*	65125	12	0.4	Serine/threonine kinase, mitotic kinase
*RTEL1*	51750	20	0.37	ATP-dependent DNA helicase (DNA repair)
*IGF1R*	3480	15	0.36	Receptor tyrosine kinase
*FGFR3*	2261	4	0.3	Receptor tyrosine kinase
*CDK6*	1021	7	0.3	Serine/threonine-protein kinase, cell cycle regulator

The copy number ranges of the cancer amplified genes were analyzed in individual TCGA patient tumors to determine the extent of gene amplification (Fig. S1, S2). Some genes displayed high level amplification corresponding to 10–20 gene copies, while other genes displayed low level 3–8 copy number amplifications. The chromosome 1q amplicon, which contained *PRKAB2*, *APH1A*, *ARNT*, and *SETDB1*, showed low level amplification (3–10 copies), while the chromosome 12q amplicon, which contained *MDM2*, *YEATS4*, and *FRS2*, showed high level amplification (10–20 copies) (Fig. S1, S2). Other genes with high level amplifications include *PRKAB2* (6–10 copies in ovarian cancer), *MDM4* (10–30 copies in glioblastoma), *MDM2* (10–15 copies in lung adenocarcinoma), *PIK3CA* (5–20 copies in lung squamous cancer), *DCUN1D1* (5–15 copies in lung squamous cancer), *FADD* and *PPFIA1* (each with 5–10 copies in head and neck cancer), *NDUFC2* (5–15 copies in ovarian cancer), and *RAP1B* (5–15 copies in lung adenocarcinoma). MAP-kinase associated genes also showed high level amplification, with receptor tyrosine kinases *ERBB2*, *IGF1R*, and *EGFR* all highly amplified, as expected. The MAP kinase adaptor proteins *FRS2* and *GRB7* were also highly amplified (10–20 copies in lung adenocarcinoma and breast cancer, respectively). Cell cycle regulators, such as *CCNE1* (10–20 copies in ovarian cancer), were also highly amplified, as expected. In addition to copy number ranges, the frequency of gene amplification in patient tumors was calculated by using copy number 4 as a cutoff for amplification (Fig. S4). A significant number of genes were amplified in greater than 30 percent of cancer patients, including *DCUN1D1* (43% of lung squamous cancers), *FADD* and *PPFIA1* (∼30% of head and neck cancers), and *PRKCI* (36% of lung squamous cancers) (Fig. S4). While amplification was the primary genomic change for these genes, a number of genes also carried somatic mutations, such as *PIK3CA*, *KRAS* and *NOTCH2*. In these cases, the amplifications and mutations were largely mutually exclusive (Fig. S4).

### MAPK pathway amplified genes

The 73 cancer amplified genes were further analyzed by shRNA validation to verify cancer driver activity. Project Achilles is a large scale effort to catalogue genetic vulnerabilities in cancer cell lines by using a genome-wide shRNA library to identify genes that affect cancer cell survival/proliferation [11]. We mined the Achilles database to determine which of the 73 cancer amplified genes may play a role in cancer cell survival/proliferation. The Achilles library is comprised of multiple shRNA hairpins and we calculated a composite shRNA score based on the effects of multiple lentiviral shRNA hairpins on infected cancer cell lines. Genes demonstrating a low shRNA score in infected cell lines are presumed to be important for cancer cell survival and may represent putative cancer drivers. The shRNA scores are only valid when multiple shRNA hairpins consistently demonstrate cancer cell inhibition (termed “large correlation”). The Achilles database was queried with the 73 genes and those genes with “large correlation” shRNA activity were identified, and their shRNA scores were calculated across several hundred cancer cell lines (Fig. S3). Several genes had negative shRNA scores across most of the cancer cell lines and were presumably critical for cancer cell survival/proliferation. These gene included *KRAS*, *PRKAB2*, *GRB7*, *BRD4*, *PRPF6*, *BCL9*, *PPFIA1* and *NOTCH2*. Other genes showed negative shRNA scores in a subset of the cancer cell lines, such as *CCND1*, *NDUFC2*, *YEATS4*, *GTPBP4*, and *CHD1L* (Fig. S3). In these cases, further validation with siRNA or shRNA is required to verify inhibition of cancer cell proliferation or survival.

**Figure 5:**
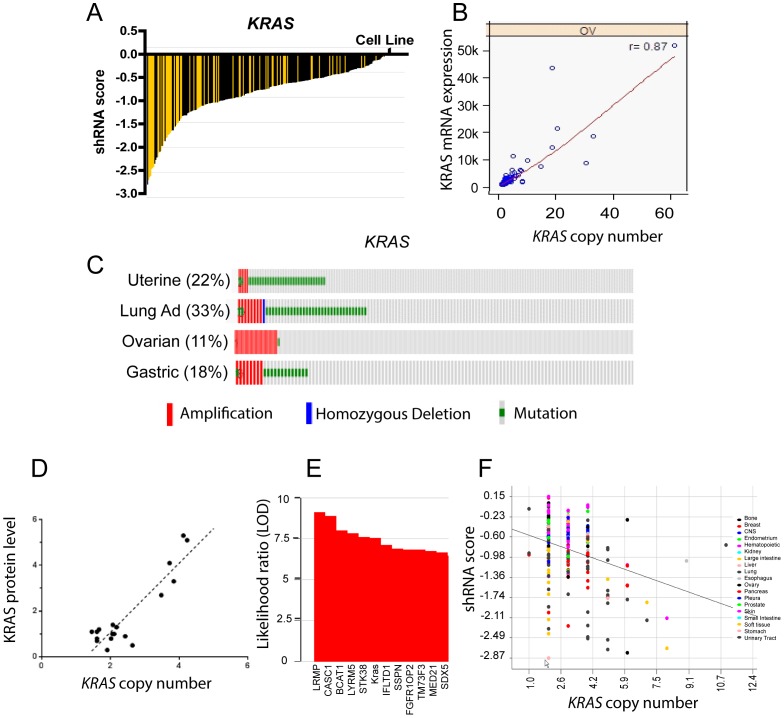
Cancer amplified genes in the MAP kinase pathway. (A) *KRAS* shRNA activity in a panel of cancer cell lines (Project Achilles). shRNA score denotes the log2 based decrease in *KRAS* shRNA compared to pooled shRNA in cancer cell lines after several rounds of proliferation post-shRNA infection [11]. A negative shRNA score suggests decreased cancer cell proliferation/survival after shRNA transfection. Yellow bars indicate cell lines with *KRAS* copy number >4 and black bars indicate cell lines with *KRAS* copy number <4. (B) Copy number (x-axis) and mRNA expression (y-axis) for *KRAS* in a panel of ovarian cancers. Correlation coefficient for copy number and mRNA expression are listed in the top right (r value). (C) Frequency of amplification (red bar), mutation (green bar), and deletion (blue bar) for *KRAS* in various cancers. The percentages shown reflect the overall rate of gene amplification, mutation and/or deletion in each cancer type. Vertical aligned bars reflect samples from the same patient. (D) *KRAS* copy number (x-axis) and KRAS relative protein level (y-axis) as measured by western blot in a panel of lung cancer cell lines grown in vitro. (E) Gene amplifications associated with sensitivity to *KRAS* shRNA in cancer cell lines (Project Achilles). Y-axis = Log10 Likelihood Ratio (LOD) of gene amplification being associated with shRNA score by comparing each gene amplification model to the “null model” without any gene amplification. (F) *KRAS* copy number (x-axis) and *KRAS* shRNA score (y-axis) for individual cancer cell lines color-coded by tumor type (data obtained from Project Achilles). Trendline shown for mean values in each copy number bin.

The 73 cancer amplified genes included a number of receptor tyrosine kinases, GTPases, adaptors and signaling genes in the MAP kinase pathway. One of the most important amplified genes is the proto-oncogene *KRAS*, a small GTPase that is frequently mutated in lung, pancreatic and colorectal cancers [24]. A single amino acid substitution in *KRAS* results in activating mutation and dependence of the cancer cells on the MAP kinase pathway. Although mutation of *KRAS* is frequently used for cancer diagnosis and clinical management, *KRAS* amplification is typically not tested in patients. Our data suggests that *KRAS* is amplified in ovarian, gastric, lung adenocarcinoma, and uterine cancers, with a copy number range 10–40 in ovarian cancers (Figure 5B, 5C). *KRAS* mutations and amplifications are largely mutually exclusive in uterine, gastric, and lung cancers (Figure 5C). Interestingly, 11 percent of ovarian cancers display *KRAS* amplification (copy number 4 or greater) and these tumors largely do not have *KRAS* mutation. To determine if *KRAS* amplification had functional consequence in cancer, we queried Project Achilles and found that most cancer cell lines displayed a negative shRNA score in response to *KRAS* shRNA infection (Figure 5A). *KRAS* copy number was negatively correlated with shRNA score, suggesting that cancer cell lines with *KRAS* amplification are most sensitive to Kras shRNA-mediated cell death (Figure 5A, 5F). Indeed, *KRAS* copy number was among the best predictors of sensitivity to *KRAS* shRNA (Figure 5E). *KRAS* copy number was also positively correlated to *KRAS* protein levels in a panel of cancer cell lines (Figure 5D). These data suggest that amplification of wild-type *KRAS* may be an independent cancer driver in several disease subtypes.

**Figure 6:**
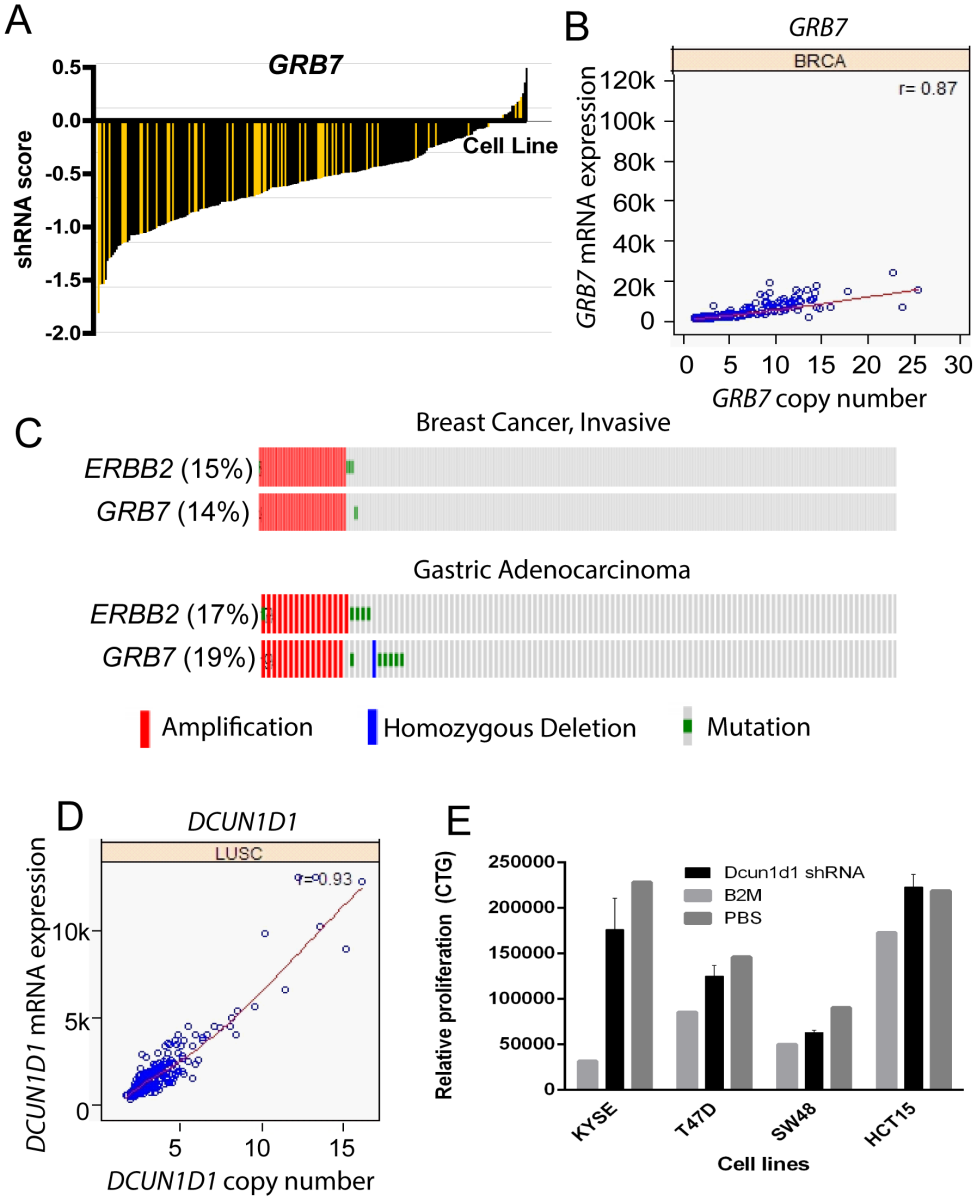
*GRB7* and *DCUN1D1* are novel cancer amplified genes with putative driver activity. (A) *GRB7* shRNA activity in a panel of cancer cell lines (Project Achilles). shRNA score denotes the log2 based decrease in *GRB7* shRNA compared to pooled shRNA in cancer cell lines after several rounds of proliferation post-shRNA infection [11]. A negative shRNA score suggests decreased cancer cell proliferation/survival after shRNA transfection. Yellow bars indicate cell lines with *GRB7* copy number >4 and black bars indicate cell lines with *GRB7* copy number <4. (B) Copy number (x-axis) and mRNA expression (y-axis) for *GRB7* in a panel of breast cancers. Correlation coefficient for copy number and mRNA expression are listed in the top right (r value). (C) Frequency of amplification (red bar), mutation (green bar), and deletion (blue bar) for *GRB7* and *ERBB2* in various cancers. The percentages shown reflect the overall rate of gene amplification, mutation and/or deletion in each cancer type. Vertical aligned bars reflect samples from the same patient. (D) Copy number (x-axis) and mRNA expression (y-axis) for *DCUN1D1* in lung squamous cancers. Correlation coefficient for copy number and mRNA expression is listed in the top right (r value). (E) Relative proliferation (y-axis) of cancer cell lines KYSE, T47D, SW48, and HCT15 cells 6 days after infection with *DCUN1D1* lentiviral shRNA particles, as measured by Cell Titer Glo assay.

A number of MAP kinase adaptor genes were also amplified in TCGA datasets, including the FGFR adaptor *FRS2* and the EGFR family adaptor *GRB7*. *GRB7* displayed an Achilles shRNA hairpin profile similar to *KRAS* (Fig. 6A), suggesting that *GRB7* may be necessary for cancer cell survival/proliferation. *GRB7* is amplified in a chromosome 17q amplicon with *PPP1R1B* and *ERBB2* and displays a 10–25 copy number range (Fig. 6B). Interestingly, *GRB7* and *ERBB2* are co-amplified in 15% of invasive breast cancers and 17–19% of gastric adenocarcinomas (Figure 6C). Since *GRB7* is a molecular adaptor for EGFR receptor tyrosine kinases, including *ERBB2*, the amplification of *GRB7* may have a functional consequence in Her-2 driven cancers. In Project Achilles, both the *GRB7* and *ERBB2* composite Achilles scores showed a statistically signification association with *GRB7* amplification (p = 0.001 and 0.0009, respectively, data not shown), indicating that *GRB7* may be necessary for cancer cells harboring this amplicon, as previously suggested [25]. Recent reports suggest that *ERBB2/GRB7* co-amplification may be a necessary step for cancer progression in specific cancer types, such as Barrett's carcinoma [26]. Further, *GRB7* amplification may be a drug resistance mechanism during anti-Her-2 therapy, such as lapatinib treatment in breast cancers [27].

### *DCUN1D1* as a novel amplified cancer gene

We identified a number of novel cancer targets amplified in TCGA datasets. Among the list was *DCUN1D1*, which was amplified in 43% of lung squamous cancers (copy number 4 or greater cutoff) and displayed a 5–15 copy number range (Figure 6D, Fig. S4). *DCUN1D1*, also known as squamous cell carcinoma related oncogene (*SCCRO*), is an E3 ubiquitin ligase component required for neddylation and it has been linked to the Hedgehog pathway [28]. *DCUN1D1* amplification in squamous cancers is associated with poor outcome and its knockdown in cells by shRNA leads to apoptosis. The overexpression of *DCUN1D1* in cell lines is sufficient to induce carcinogenesis in vitro and in vivo, suggesting that *DCUN1D1* is a putative oncogenic driver [28]. We further validated *DCUN1D1* oncogenic activity through shRNA knockdown in the *DCUN1D1*-amplified cell lines KYSE and T47D and the wild-type cell lines HCT15 and SW48. On average, the *DCUN1D1*-amplified cell lines showed reduced cell proliferation after six days treatment with *DCUN1D1* shRNA relative to the control cells (Figure 6E). These data suggest that *DCUN1D1* may be a novel oncogenic driver amplified in squamous cancers, in particular lung squamous cancer. Further studies will be necessary to explore its link to the Hedgehog pathway and its feasibility as a therapeutic target.

### Epigenetic regulators amplified in TCGA datasets

**Figure 7:**
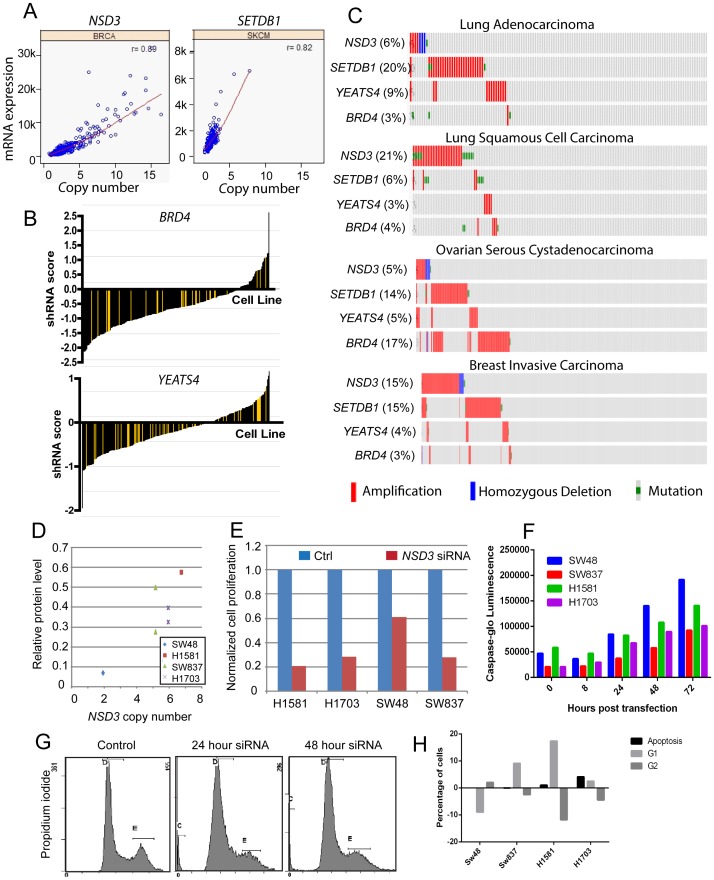
Epigenetic regulatory genes as putative cancer amplified driver genes. (A) Copy number (x-axis) and mRNA expression (y-axis) for *NSD3* and *SETD1* in breast cancers and melanomas, respectively. Correlation coefficient for copy number and mRNA expression are listed in the top right (r value). (B) *BRD4* and *YEATS4* shRNA activity in a panel of cancer cell lines (Project Achilles). shRNA score denotes the log2 based decrease in the representative shRNA compared to pooled shRNA in cancer cell lines after several rounds of proliferation post-shRNA [11]. Yellow bars indicate cell lines with *BRD4* or *YEATS4* copy number >4 and black bars indicate cell lines with *BRD4* or *YEATS4* copy number <4. (C) Frequency of amplification (red bar), mutation (green bar), and deletion (blue bar) for *NSD3*, *SETDB1*, *YEATS4*, and *BRD4* in various cancers. The percentages shown reflect the overall rate of gene amplification, mutation and/or deletion in each cancer type. Vertical aligned bars reflect samples from the same patient. (D) Relative NSD3 protein level (y-axis, normalized to b-actin protein levels) compared with *NSD3* copy number (x-axis) in SW48, H1581, SW837, and H1703 cells. (E) Relative proliferation (y-axis) and (F) relative apoptosis levels of cancer cell lines H1581, H1703, SW48, and SW837 cells 3 days after transfection with *NSD3* siRNA, as measured by Cell Titer Glo and Caspase Glo assays, respectively. (G) Cell cycle profile of H1703 cells 24 or 48 hours after transfection with *NSD3* siRNA compared to non-transfected controls. (H) Relative changes of cells in apoptosis, G1 or G2 phases (y-axis) in cell lines 48 hours-post *NSD3* siRNA transfection compared to uninfected controls.

Chromatin modifying genes and epigenetic regulators are often mutated in cancer patients and our data suggest that these genes are also amplified in many cancer types. In particular, we identified the epigenetic regulatory genes *NSD3*, *SETDB1*, *YEATS4*, and *BRD4* as putative amplified cancer drivers in several cancer types. The copy number ranges for these genes varied widely, with *NSD3*/*WHSC1L1* amplified at high copy number levels (5–15 copies) in breast cancer patients, while *SETDB1* was amplified at 3–6 copy range in melanoma (Figure 7A). We further mined the Achilles shRNA data to determine if these genes had a cancer driver signature. While *SETDB1* and *NSD3* did not have correlating hairpins to carry out the analysis, we did observe that *MCL1* shRNA composite score correlated well with *SETDB1* amplification (data not shown), suggesting that 1q21 amplification may signal dependence on *SETDB1* and *MCL1*. The epigenetic reader *BRD4* exhibited multiple correlating hairpins that carried negative shRNA scores in cancer cell lines (Figure 7B). In contrast, the histone acetyltransferase *YEATS4* displayed negative shRNA scores in a subset of cancer cell lines (Figure 7B). We further analyzed the frequency of amplification of these four epigenetic genes across cancer subtypes. The relative amplification of the genes varied substantially across the cancer types, with *SETDB1* amplified in 20% of lung adenocarcinomas, *NSD3* amplified in 21% of lung squamous cancers, and *BRD4* amplified in 17% of ovarian serious adenocarcinomas (Figure 7C). In lung squamous cancers and adenocarcinomas, the amplifications of the four genes were largely mutually exclusive. Invasive breast cancers were divided into two largely mutually exclusive groups with amplifications in *NSD3* (15%) and *SETDB1* (15%). These data suggest that distinct epigenetic regulators may control specific cancer disease subtypes.

While *SETDB1* has been validated as a cancer driver in several disease types [19], [20], *NSD3*/*WHSC1L1* has not been widely investigated for its role in oncogenesis and tumor progression. We identified cancer cell lines with *NSD3* amplification for further experimental validation, including the non small-cell lung cancer (NSCLC) cell lines H1581 (7 copies Nsd3) and H-1703 (6 copies Nsd3), as well as the colorectal cancer cell line SW837 (5 copies Nsd3). The colorectal cancer cell line SW48 was used as a non-*NSD3*-amplified control. NSD3 protein was detected by western blot in the four cancer cell lines, and the relative NSD3 protein levels positively correlated with *NSD3* copy number (Figure 7D), suggesting that *NSD3* amplification leads to higher NSD3 protein levels. We evaluated the consequence of *NSD3* depletion in cancer cells through siRNA knockdown. Using the Cell Titer Glo cell proliferation assay, we found that *NSD3* siRNA knockdown led to reduced cancer cell proliferation in all four cell lines, and the relative inhibition of proliferation correlated with *NSD3* copy number (e.g., 80% inhibition in H1581 cells versus 40% inhibition in SW48 cells) (Figure 7E). To determine if the effects of *NSD3* knockdown were due to changes in proliferation or cell survival, we measured the levels of apoptosis in cancer cells after *NSD3* siRNA transfection using the Caspase Glo assay. Interestingly, all four cancer cell lines exhibited apoptosis starting 24 hours after *NSD3* siRNA transfection, and the relative apoptosis levels increased steadily after 48 and 72 hours post-transfection (Figure 7F). This suggested that loss of *NSD3* lead to apoptosis of cancer cells, suggesting that *NSD3* may be a bona fide cancer driver gene. A recent report also found that *NSD3* knockdown led to reduced cell proliferation and increased apoptosis, which was attributed to G2/M cell cycle arrest [15]. The authors suggested that *NSD3* played a role in regulating the transcription of cell cycle genes, specifically *CCNG1* and *NEK7*. We measured the relative fraction of cancer cells in G1, S and G2 phases following *NSD3* siRNA transfection. We did not observe an increase in G2-phase cells after *NSD3* siRNA transfection (Figure 7G). In fact, there were fewer cells in G2 phase and more cells in G1 phase after *NSD3* knockdown (Figure 7H). These data suggest that *NSD3* may be a bona fide amplified driver gene in multiple cancer types but further work will be necessary to define its precise mechanism of action in cancer.

## Discussion

We carried out a GISTIC2 analysis of gene amplifications in TCGA datasets and identified a number of amplified genes with cancer driver activity. The initial bioinformatics screen yielded 461 genes with statistically significant amplification in 2 or more TCGA datasets, and subsequent screening yielded 73 potentially druggable amplified genes with known or putative roles in oncogenesis. Among the 73 genes were a number of established cancer driver genes and validated drug targets, including *ERBB2*, *EGFR* and *PIK3CA*. Since the majority of the genes were clustered in amplicons throughout the genome, we further calculated the “cancer driver score” for each gene by calculating the correlation between its copy number and mRNA expression in each TCGA cancer subtype. A chromosome 1q cluster with 12 amplified genes contained 4 genes with overall copy number versus mRNA expression correlation greater than 0.5, and the two most highly ranked genes in the amplicon were *SETDB1* and *APH1A*. Interestingly, *SETDB1* was recently identified as an important amplified cancer driver gene in lung cancer and melanoma [19], [20]. Further, the Notch pathway is an important driver in oncogenesis, as activating mutations in Notch pathway components, such as *NOTCH1* and *NOTCH2*, can drive specific cancer types [29]. *APH1A* is a gamma-secretase complex subunit in the Notch pathway and its amplification may be an important patient tailoring marker for anti-Notch therapeutics. We should note that while some cancer amplified genes had a low overall copy number versus mRNA expression correlation, many genes had high cancer subtype-specific copy number versus mRNA expression correlation. For instance, while *PDGFRA* had an overall copy number versus mRNA expression correlation of 0.12 and was not included in the 40 genes with a “cancer driver signature,” it showed high copy number versus mRNA expression correlation in glioblastoma (r = 0.8) and lung squamous cancer (r = 0.7) (Table 1). This suggests that cancer amplified genes should be investigated in the disease subtype(s) in which they may play important driver roles.

The bioinformatics tools described here can be utilized for prioritizing candidate genes for drug discovery and identifying cancer subtypes for patient tailoring. Analysis of TCGA datasets was used to identify cancer subtypes containing gene amplifications of interest. Project Achilles and siRNA knockdown can be used to validate the role of a gene amplification in a specific cancer disease subtype. Additional experiments with candidate lead compounds and siRNA/shRNA knockdown reagents can be used to further validate the cancer driver activity of a gene amplification in a specific disease context. Further, siRNA knockdown of the candidate gene in normal cells can be used to assess potential toxicity of a therapeutic candidate. Development of a candidate therapeutic can also be aided by structural determination of the protein products of gene amplification. These tools can be combined to prioritize candidate genes for drug discovery efforts.

### Metabolic cancer amplified genes

Sub-group analysis of the 73 cancer genes amplified in TCGA datasets identified a large number of gene families with diverse roles in oncogenesis. Several amplified genes were linked to metabolic pathways, including the NADH dehydrogenase subunit *NDUFC2* and the AMP-activated protein kinase subunit *PRKAB2*. Cancer cells utilize distinct energy production mechanisms compared to normal cells, such as the high rate of aerobic glycolysis (termed the Warburg effect), and these mechanisms can be utilized to diagnose and treat cancers [30]. *NDUCF2* is an accessory subunit in mitochondrial complex I, an important component of the mitochondrial respiratory chain that catalyzes NADH oxidation and produces ATP. Disruptions in complex I have been linked to cancer initiation/progression due to alterations in the NAD-/NADH ratio [31]. Mitochondrial complex I inhibitors can induce cell death and autophagy, possibly mediated through reactive oxygen species [32]. *NDUFC2*-amplified cancer cells may have alterations in mitochondrial energy metabolism, but further work will be necessary to investigate these mechanisms and potential sensitivity to complex I inhibitors. The AMP-activated protein kinase (AMPK) is another important metabolic sensor that regulates metabolic pathways, including fatty acid biosynthesis and glycolysis, and promotes cancer cell survival [33]. *PRKAB2* is a regulatory subunit in the AMPK complex and is overexpressed in several cancers, including ovarian cancers [34]. Interestingly, it was recently suggested that the AMPK promotes tumor cell survival primarily through the regulation of NADPH homeostasis via fatty acid oxidation and inhibition of acetyl-CoA carboxylases [35]. This mechanism allows cancer cells to maintain NADPH levels under high stress conditions, such as hypoxia and anchorage-independent growth. Further work will be necessary to study the role of AMPK in *PRKAB2* amplified cancer cells and to characterize the role of *PRKAB2* as a putative cancer driver gene.

### Epigenetic cancer amplified genes

A number of chromatin-modifying genes and chromatin reader genes were amplified in TCGA datasets, including the genes *SETDB1*, *NSD3*, *YEATS4*, and *BRD4*. Epigenetic regulatory genes comprise a large family of genes that add, modify or read modifications on DNA and histones. These modifications can lead to abnormal alterations in gene transcription, replication or repair, which can lead to the induction and maintenance of many cancers [36]. At least four distinct DNA modifications and 16 classes of histone modifications have been recognized [37]. A large number of DNA/chromatin modifying enzymes directly conjugate these modifications to target DNA/histones, including *SETDB1* (a H3K9 methyltransferase), *NSD3* (a H3K4/H3K27 methyltransferase) and *YEATS4* (histone acetyltransferase). Other chromatin “reader” genes, such as *BRD4*, recognize these DNA/histone modifications and recruit additional chromatin modifier/remodeling enzymes to the sites [37]. Together these epigenetic regulatory genes play important roles in cancer initiation/progression, with a number of cancers being driven by mutations in the gene families. This includes the histone methyltransferase *MLL2* and the histone demethylases *UTX*, which are mutated in a number of cancers [38]. A number of chromatin regulatory genes have been implicated in cancer progression, specifically in the reprogramming of cancer metastases in distant organs [39]. Small molecule inhibitors have been developed for a number of these epigenetic regulatory genes and are currently under clinical development. Recently, small molecular inhibitors of *BRD4* have been identified and may hold promise for the treatment of cancer subtypes, including acute myeloid leukemia [40], [41]. The cancer driver signatures for *SETDB1*, *YEATS4*, *NSD3*, and *BRD4* in TCGA datasets suggests that these genes are important cancer therapeutic targets and potential patient tailoring markers for epigenetics drug discovery efforts.

While the amplifications of *SETDB1* and *BRD4* have been previously identified and their roles in cancer have been well-studied [19], [20], less is known about the functions of *YEATS4* and *NSD3* in cancer. The nuclear receptor binding SET domain (NSD) protein family is made of three histone methyltransferases – *NSD1*, *NSD2*/*MMSET*/*WHSC1*, and *NSD3*/*WHSC1L1*. *NSD1* has been linked to several cancers, including multiple myeloma and lung cancer, and translocations involving *NSD1* and *NUP98* have been identified in childhood acute myeloid leukemia [42]. The *NUP98*-*NSD1* fusion protein is an active H3K36 methylase, suggesting that *NSD1* enzymatic activity is a necessary oncogenic driver for this cancer. It has been suggested that overexpressed *NSD1* acts as an oncogene by activating genes that are normally silenced by H3K27 methylation [43]. *NSD2* also has been linked to several cancers, such as prostate cancer and multiple myeloma, and it is also the target of translocations in multiple myeloma [42]. *NSD3* is overexpressed in a number of cancers, including breast cancer and lung cancer, but its role in oncogenesis has not been widely investigated. Among the 73 cancer amplified genes we identified in this study, *NSD3* was the most highly ranked gene in terms of putative cancer driver activity. It showed high copy number versus mRNA expression correlation in multiple cancer types, including bladder, breast, liver, lung, ovarian, head and neck, and colorectal cancers. *NSD3* knockdown by siRNA led to reduced cell proliferation and increased apoptosis in *NSD3*- amplified cell lines. The mechanism of *NSD3*-siRNA-mediated cell death did not appear to involve cell cycle regulation, as reported previously, and further work will be necessary to define its precise role in oncogenesis [15]. Our data suggest that *NSD3* is a compelling drug target for cancer and that *NSD1*/*NSD2*/*NSD3* structural similarity should be used for structure-based drug design to develop a new class of histone methyltransferase inhibitors for cancer.

### Ubiquitin-like modifiers

Post-translational modifications of proteins by ubiquitin and ubiquitin-like proteins have emerged as important regulators of cancer cell signaling, survival, and homeostasis. Ubiquitylation of proteins was originally described as a “destruction tag” for defective proteins, but recent studies have identified important roles for ubiquitylation in many hallmarks of cancer, such as the cell cycle, DNA repair, and apoptosis [44]. Other ubiquitin-like protein modifications exist and can similarly regulate oncogenesis, such as sumoylation (SUMO tag), neddylation (NEDD8 tag), ISGylation (ISG15 tag), and fatylation (FAT10 tag). Ubiquitin and ubiquitin-like ligases are key regulators of these small protein modifications and they have emerged as important oncogenes and tumor suppressors in many cancer types [44]. The ubiquitin ligases *CBL* and *SKP2* were identified as candidate oncogenes, while the ubiquitin ligase *FBXW7* is a bona fide tumor suppressor that is mutated frequently in breast and colorectal cancers [3]. We have identified a number of ubiquitin and ubiquitin-like ligases that are amplified in TCGA datasets and show evidence of cancer driver activity, including *MDM2*, *DCUN1D1* and *PIAS3*. Of these genes, the best characterized is *MDM2*, a E3 ubiquitin ligase that mediates p53 polyubiquitylation and degradation, allowing for silencing of p53 in p53-wild type cancer cells [45]. Transgenic mouse model studies showed that *MDM2* overexpression was sufficient to induce carcinomas and lymphomas, an effect that depended on p53 inhibition [45]. We also identified *MDM4* as a cancer amplified gene with potential cancer driver activity. Like *MDM2*, *MDM4* inhibits p53 but its mechanism relies on direct binding to the p53 transactivation domain (rather than ubiquitin-mediated degradation) and binding to *MDM2* to preventing its degradation [46]. Recently, small molecular inhibitors of *MDM2* and *MDM4* have been developed and are currently being evaluated in clinical trials for several cancer types [47].

Recent studies suggest that ubiquitin-like modifiers play important roles in oncogenesis and tumor progression. A number of transcriptional cofactors and chromatin remodeling factors are targets of the small ubiquitin-like modifier (SUMO), and as a result a number of oncogenes and tumor suppressors are regulated by SUMOylation [48]. In breast cancer, the *BRCA1* DNA damage response protein is modified by the small ubiquitin-like modifier (SUMO) in response to genotoxic stress, and several SUMO E3 ligases are required for the downstream DNA damage response [49]. We identified the SUMO family member *PIAS3* as a putative cancer driver gene that is amplified in TCGA datasets. *PIAS3* is an E3 SUMO ligase that has been linked to regulation of *STAT3* and *ERBB4*, two important signaling pathways in oncogenesis [50], [51]. In addition to SUMOylation, other ubiquitin-like modifications play important roles in oncogenesis, such as neddylation, ISGylation, and fatylation [52]. We identified the neddylation ligase *DCUN1D1* as a putative amplified cancer driver that was amplified in over 40% of lung adenocarcinomas and squamous cancers. We found that *DCUN1D1*-amplified cancer cell lines exhibited decreased cell proliferation/survival in response to *DCUN1D1* knockdown, consistent with earlier reports that *DCUN1D1* knockdown leads to apoptosis in cells [28]. *DCUN1D1* overexpression in cells is sufficient to induce carcinogenesis, suggesting that it may be a bona fide cancer driver gene [28]. Further, *DCUN1D1* activity has been linked to regulation of *GLI1*, an important signaling molecule in the Hedgehog pathway. These data suggest that ubiquitin and ubiquitin-like modifiers are important regulators of oncogenesis that are amplified in the cancer genome, and further work will be necessary to evaluate the therapeutic potential of targeting these enzymes for cancer.

### MAP kinase pathway

Mitogen-activated protein kinase (MAPK) pathways are kinase modules that link extracellular signals to intracellular signaling cascades and regulate fundamental processes in oncogenesis, such as growth, proliferation, differentiation, migration, and apoptosis [53]. Activating mutations can occur at multiple levels in the pathways to drive oncogenesis. The receptor tyrosine kinases *EGFR* and *ERBB2*, the GTPases *KRAS*, *NRAS*, and *HRAS*, and the kinase *BRAF* are frequently mutated in cancers and can drive tumor proliferation. Among the most important members of the family is *KRAS*, which is mutated in over 30% of colorectal cancers and predicts poor response to anti-EGFR therapy [3], [54]. The data here suggest that in addition to being mutated in cancers, *KRAS* is also amplified in ovarian, gastric, lung adenocarcinoma, and uterine cancers, with a copy number range 10–40 in ovarian cancers. Interestingly, *KRAS* mutations and amplifications are largely mutually exclusive in these cancer types. *KRAS* amplification is currently not used as a diagnostic or clinical management marker, but its utilization may be warranted in specific cancer types. Recent studies suggest that *KRAS* gene amplification predicts resistance to anti-EGFR therapy and anti-Met therapy, suggesting that *KRAS* amplification may be a resistance mechanism to MAP kinase inhibitors [54], [55]. Interestingly, *KRAS* amplification was also found at higher frequency in endometrial cancer metastases versus primary tumors, suggesting that *KRAS* amplification may be a potential mechanism for metastasis formation and cancer progression [56]. Further work will be necessary to define the role of *KRAS* amplification in cancer metastases and in drug resistant tumors, especially in tumors that have acquired resistance to receptor tyrosine kinase inhibitors. Other MAP kinase associated genes that were amplified in TCGA datasets include the MAP kinase adaptor genes *FRS2* and *GRB7*. *GRB7* and its associated receptor tyrosine kinase *ERBB2* were co-amplified in 15–20% of breast and gastric cancers. In some cases, co-amplification of the adapter protein may be necessary for oncogenesis, while in other cases the amplification of the adapter protein may be an acquired resistance mechanism to anti-tyrosine kinase therapy [26], [27]. Further work is necessary to define the utility of *FRS2* and *GRB7* amplification as clinical drug response markers.

In summary, we have carried out a genome-wide analysis of TCGA datasets to identify amplified genes with putative cancer driver activity. The analysis was based on patient tumor-derived gene copy number and mRNA expression, siRNA/shRNA gene knockdown and association with clinical parameters. We identified a number of amplified genes with a wide range of activity in oncogenesis, consistent with the various hallmarks of cancer [57]. A number of genes are novel drug candidates for future drug development efforts, such as *NSD3*. Other genes may serve as potential diagnostic markers to predict drug response/resistance, such as *GRB7*. The TCGA efforts have advanced our understanding of cancer biology by identifying the primary genetic drivers of cancer and linking novel therapeutics to these genetic backgrounds. These advancements will help shape the future era of personalized medicine and usher in a new era of diagnosis and therapy for cancer patients.

## Supporting Information

Figure S1 **Copy number and mRNA expression values for cancer amplified genes on chromosome 1–11.** Copy number (x-axis) and mRNA expression (y-axis) are shown for each gene and the associated chromosomal location/cluster is shown at the top of each graph. Each plot represents data from a TCGA dataset/cancer subtype (shown at the top of each graph) and the correlation coefficient for copy number and mRNA expression are listed in the top right (r value). The abbreviations for each cancer subtype are shown in Figure 1.
(TIF)

Figure S2 **Copy number and mRNA expression values for cancer amplified genes on chromosome 12–20.** Copy number (x-axis) and mRNA expression (y-axis) are shown for each gene and the associated chromosomal location/cluster is shown at the top of each graph. Each plot represents data from a TCGA dataset/cancer subtype (shown at the top of each graph) and the correlation coefficient for copy number and mRNA expression are listed in the top right (r value). The abbreviations for each cancer subtype are shown in Figure 1.
(TIF)

Figure S3 **shRNA activity profiles of putative amplified driver genes across a panel of cancer cell lines (Project Achilles).** shRNA score denotes the log2 based decrease in the representative shRNA compared to pooled shRNA in cancer cell lines after several rounds of proliferation post-shRNA infection [11]. Yellow bars indicate cell lines with gene amplification (copy number >4) while black bars indicate cell lines with copy number <4. Only genes with more than 1 correlating hairpins (large correlation) were included in the figure.
(TIF)

Figure S4 **Frequency of genomic aberrations among putative cancer driver genes.** Shown are amplifications (red bar), mutations (green bar), or deletions (blue bar) of each amplified gene. Genes are organized by chromosomal location. The percentages shown reflect the overall rate of gene amplification, mutation and/or deletion in each cancer type. Vertical aligned bars reflect samples from the same patient.
(TIF)

Table S1 **Identification of gene amplification in TCGA datasets using GISTIC2 algorithm (cBio portal).**
(XLSX)
