# Correction: Identification of Druggable Cancer Driver Genes Amplified across TCGA Datasets

**DOI:** 10.1371/journal.pone.0107646

**Published:** 2014-09-02

**Authors:** The PLOS ONE Staff

## Notice of Republication

This article was republished on August 18, 2014, because two of the 16 data sets from TCGA (The Cancer Genome Atlas) included in this study, i.e. Cervical cancer (moratorium - September 27, 2014) and Pancreatic ductal adenocarcinoma (moratorium - July 30, 2015), were used contrary to the Publication Guidelines at http://cancergenome.nih.gov/publications/publicationguidelines

The authors and the TCGA agreed that a republication of the article with those data sets removed would resolve this, and the Academic Editor and PLOS staff are satisfied that the results and conclusions of the article stand without these two data sets. We are republishing the article to report the results obtained from the 14 permitted data sets.

The summary of the results in the abstract was previously:

We carried out GISTIC2 analysis of TCGA datasets spanning 16 cancer subtypes and identified 486 genes that were amplified in two or more datasets. The list was narrowed to 75 cancer-associated genes with potential “druggable” properties. The majority of the genes were localized to 14 amplicons spread across the genome. To identify potential cancer driver genes, we analyzed gene copy number and mRNA expression data from individual patient samples and identified 42 putative cancer driver genes linked to diverse oncogenic processes.

The summary of the results in the abstract is now:

We carried out GISTIC2 analysis of TCGA datasets spanning 14 cancer subtypes and identified 461 genes that were amplified in two or more datasets. The list was narrowed to 73 cancer-associated genes with potential “druggable” properties. The majority of the genes were localized to 13 amplicons spread across the genome. To identify potential cancer driver genes, we analyzed gene copy number and mRNA expression data from individual patient samples and identified 40 putative cancer driver genes linked to diverse oncogenic processes.
