# Supplementary figures and images for: Identification of Druggable Cancer Driver Genes Amplified across TCGA Datasets
Source: PLoS One. 2014 May 29;9(5):e98293. doi: 10.1371/journal.pone.0098293 (PMC4038530; doi:10.1371/journal.pone.0098293)

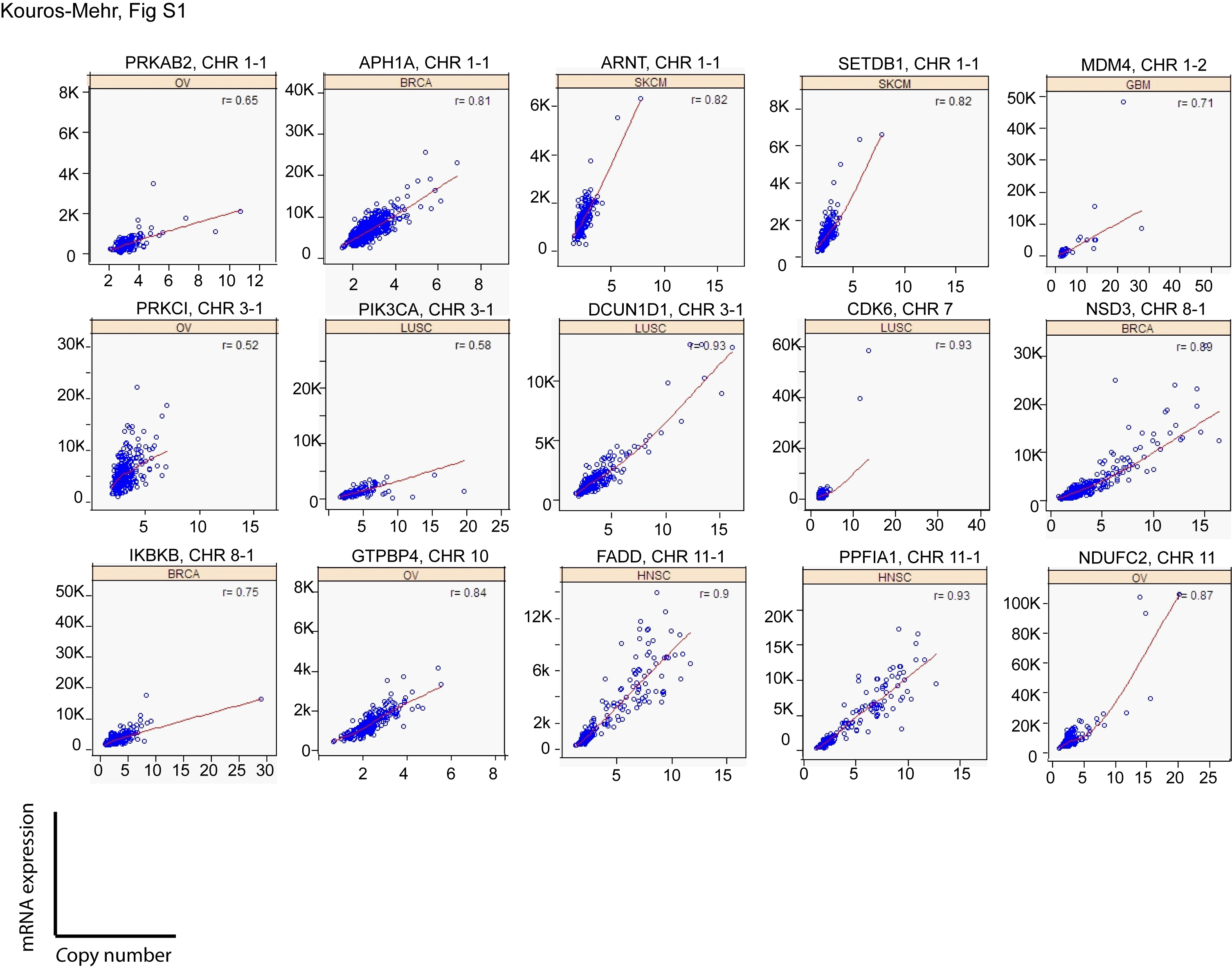

Supplement: Figure S1 — Copy number and mRNA expression values for cancer amplified genes on chromosome 1–11. Copy number (x-axis) and mRNA expression (y-axis) are shown for each gene and the associated chromosomal location/cluster is shown at the top of each graph. Each plot represents data from a TCGA dataset/cancer subtype (shown at the top of each graph) and the correlation coefficient for copy number and mRNA expression are listed in the top right (r value). The abbreviations for each cancer subtype are shown in Figure 1. (TIF) [file pone.0098293.s001.tif]

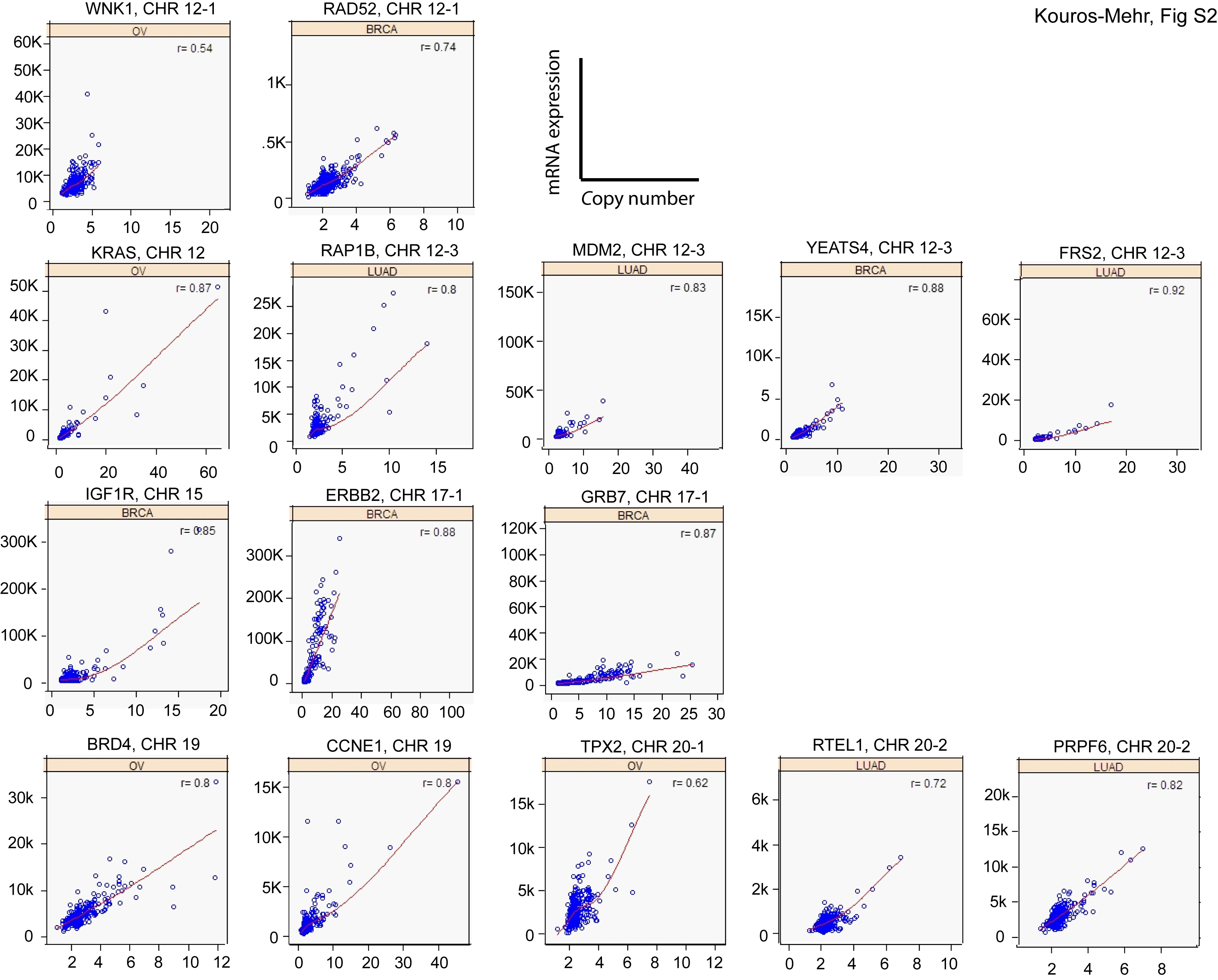

Supplement: Figure S2 — Copy number and mRNA expression values for cancer amplified genes on chromosome 12–20. Copy number (x-axis) and mRNA expression (y-axis) are shown for each gene and the associated chromosomal location/cluster is shown at the top of each graph. Each plot represents data from a TCGA dataset/cancer subtype (shown at the top of each graph) and the correlation coefficient for copy number and mRNA expression are listed in the top right (r value). The abbreviations for each cancer subtype are shown in Figure 1. (TIF) [file pone.0098293.s002.tif]

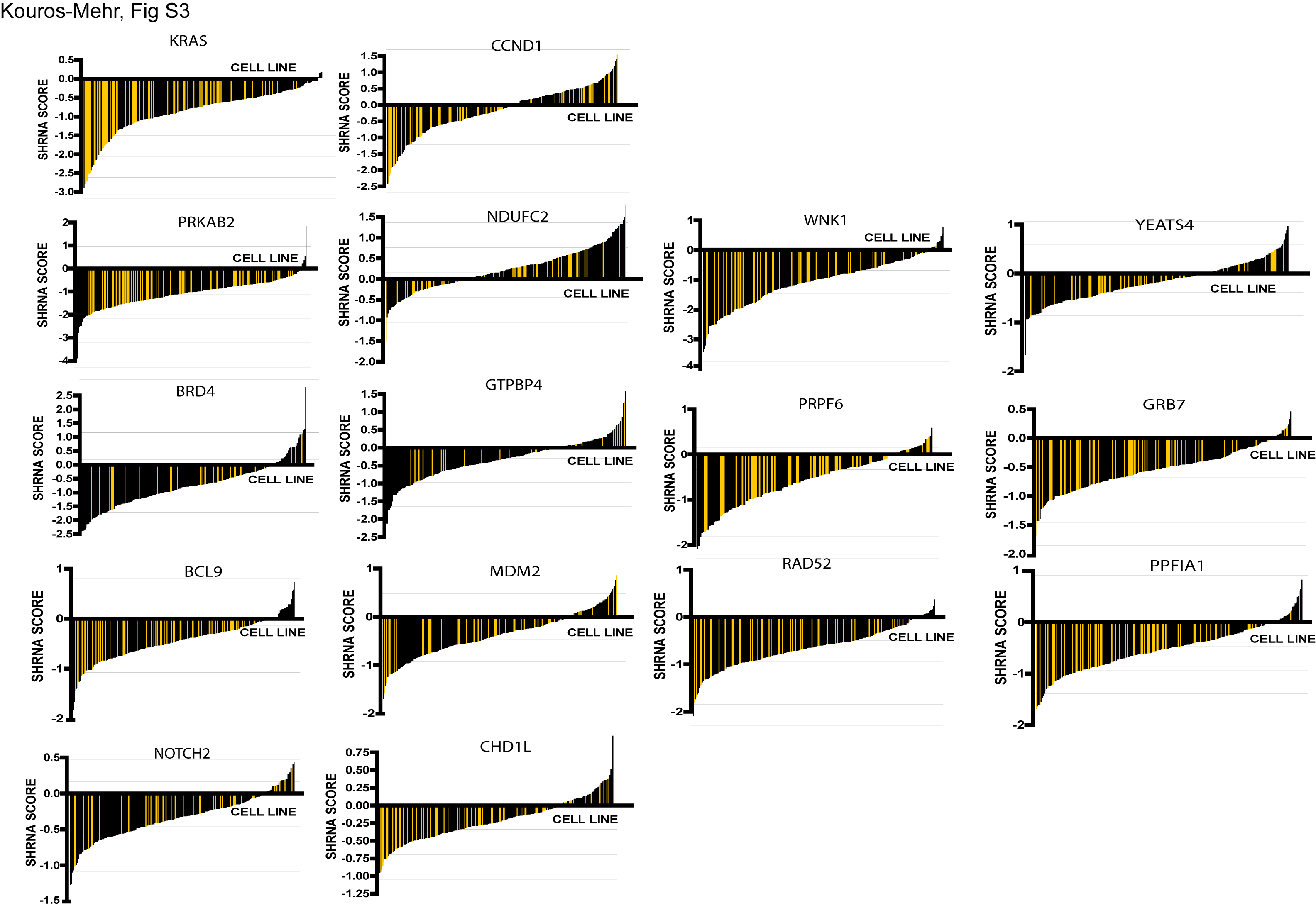

Supplement: Figure S3 — shRNA activity profiles of putative amplified driver genes across a panel of cancer cell lines (Project Achilles). shRNA score denotes the log2 based decrease in the representative shRNA compared to pooled shRNA in cancer cell lines after several rounds of proliferation post-shRNA infection [11]. Yellow bars indicate cell lines with gene amplification (copy number >4) while black bars indicate cell lines with copy number <4. Only genes with more than 1 correlating hairpins (large correlation) were included in the figure. (TIF) [file pone.0098293.s003.tif]

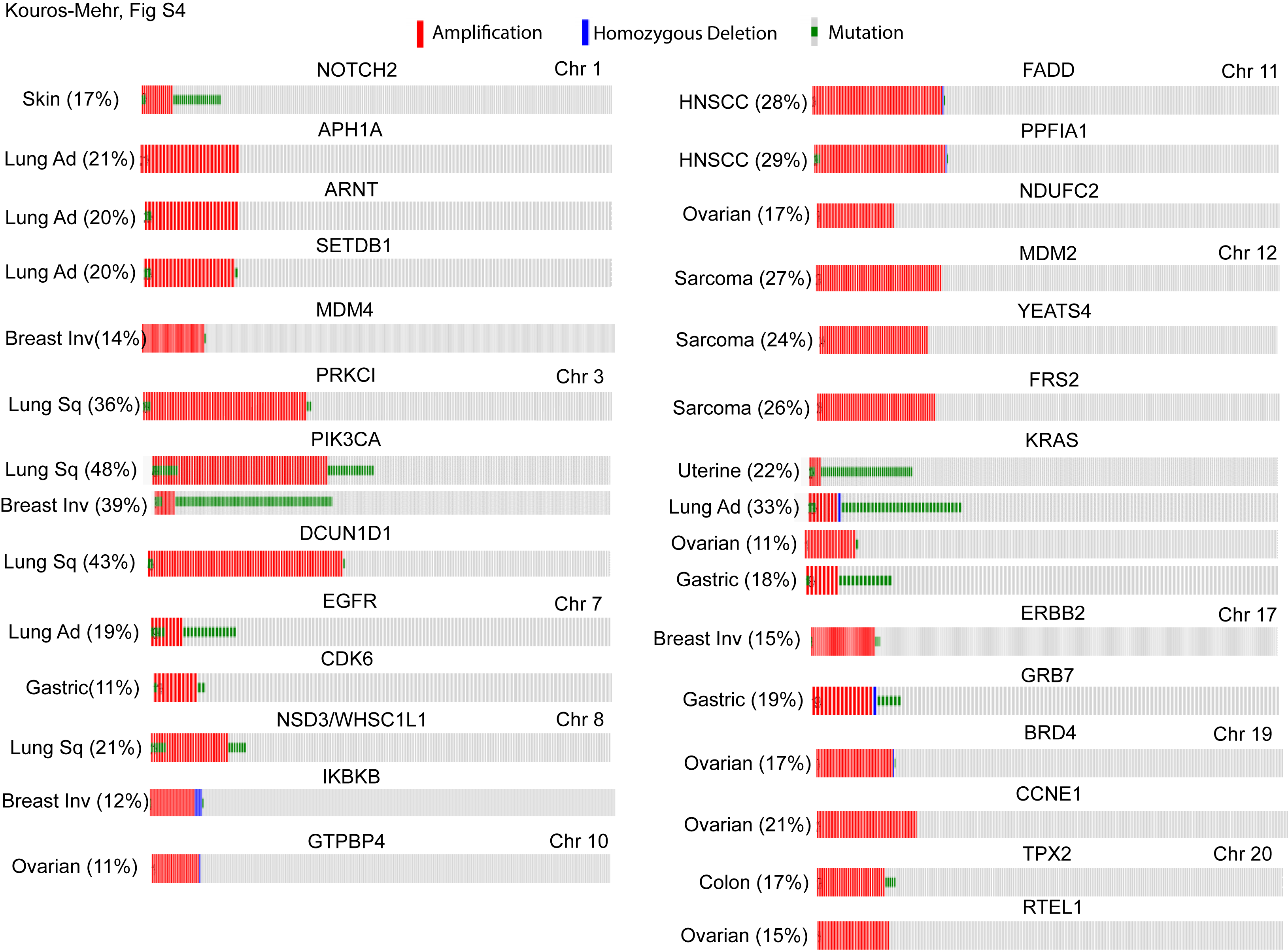

Supplement: Figure S4 — Frequency of genomic aberrations among putative cancer driver genes. Shown are amplifications (red bar), mutations (green bar), or deletions (blue bar) of each amplified gene. Genes are organized by chromosomal location. The percentages shown reflect the overall rate of gene amplification, mutation and/or deletion in each cancer type. Vertical aligned bars reflect samples from the same patient. (TIF) [file pone.0098293.s004.tif]
